# Unveiling the ventral morphology of a rare early Cambrian great appendage arthropod from the Chengjiang biota of China

**DOI:** 10.1186/s12915-024-01889-y

**Published:** 2024-04-29

**Authors:** Michel Schmidt, Xianguang Hou, Huijuan Mai, Guixian Zhou, Roland R. Melzer, Xilin Zhang, Yu Liu

**Affiliations:** 1 Yunnan Key Laboratory for Palaeobiology, Yunnan University, 2 North Cuihu Road, Kunming, 650091 People’s Republic of China; 2 MEC International Joint Laboratory for Palaeobiology and Palaeoenvironment, Yunnan University, 2 North Cuihu Road, Kunming, 650091 People’s Republic of China; 3 Bavarian State Collection of Zoology, Bavarian Natural History Collections, Münchhausenstrasse 21, 81247 Munich, Germany; 4 Faculty of Biology, Biocenter, Ludwig-Maximilians-University Munich, Großhaderner Str. 2, Planegg-Martinsried, 82152 Germany; 5 GeoBio-Center, Ludwig-Maximilians-University Munich, Luisenstrasse 37, Munich, 80333 Germany; 6 Chengjiang Fossil Museum of the Management Committee of the Chengjiang World Heritage Fossil Site, Yuxi, People’s Republic of China; 7 Southwest United Graduate School, Kunming, 650091 People’s Republic of China

**Keywords:** Cambrian fossils, Chengjiang euarthropods, Drishti, Great appendages, *Tanglangia longicaudata*, Ventral morphology, Yohoiidae

## Abstract

**Background:**

The early Cambrian arthropod clade Megacheira, also referred to as great appendage arthropods, comprised a group of diminutive and elongated predators during the early Palaeozoic era, around 518 million years ago. In addition to those identified in the mid-Cambrian Burgess Shale biota, numerous species are documented in the renowned 518-million-year-old Chengjiang biota of South China. Notably, one species, *Tanglangia longicaudata*, has remained inadequately understood due to limited available material and technological constraints. In this study, we, for the first time, examined eight fossil specimens (six individuals) utilizing state-of-the-art *μ*CT and computer-based 3D rendering techniques to unveil the hitherto hidden ventral and appendicular morphology of this species.

**Results:**

We have identified a set of slender endopodites gradually narrowing distally, along with a leaf-shaped exopodite adorned with fringed setae along its margins, and a small putative exite attached to the basipodite. Our techniques have further revealed the presence of four pairs of biramous appendages in the head, aligning with the recently reported six-segmented head in other early euarthropods. Additionally, we have discerned two peduncle elements for the great appendage. These findings underscore that, despite the morphological diversity observed in early euarthropods, there exists similarity in appendicular morphology across various groups. In addition, we critically examine the existing literature on this taxon, disentangling previous mislabelings, mentions, descriptions, and, most importantly, illustrations.

**Conclusions:**

The *μ*CT-based investigation of fossil material of *Tanglangia longicaudata*, a distinctive early Cambrian euarthropod from the renowned Chengjiang biota, enhances our comprehensive understanding of the evolutionary morphology of the Megacheira. Its overall morphological features, including large cup-shaped eyes, raptorial great appendages, and a remarkably elongated telson, suggest its potential ecological role as a crepuscular predator and adept swimmer in turbid waters.

**Supplementary Information:**

The online version contains supplementary material available at 10.1186/s12915-024-01889-y.

## Background

Cambrian arthropods bearing elongate frontal appendages with multi-annulated elements, referred to as “great appendages” have a long research history [1–3]. Besides the famous Radiodonta including anomalocarids [4–6] and hurdiids [7, 8], another group is known to also include smaller euarthropods with the so-called “great appendages” with fewer elements—the Megacheira [9]. This group contains species that are assigned to *Leanchoilia* sp. [10–12] or *Alalcomenaeus* sp. [13], whose “great appendages” bear a short peduncle that consists of a smaller number of elements than those of radiodonts, but with long and filamentous distal elements. Other great appendage euarthropods bear a similar number of pointed elements in the great appendages, but without the long, filamentous distal parts. Those include *Jianfengia* sp. [14], *Fortiforceps* sp. [9], *Yohoia* sp. [15], and *Haikoucaris* sp. [16]. Among these, there is one understudied species having an overall morphology similar to the aforementioned, but bearing a long, elongate, and slender telson–*Tanglangia longicaudata* Luo et Hu, 1999.

**Fig. 1:**
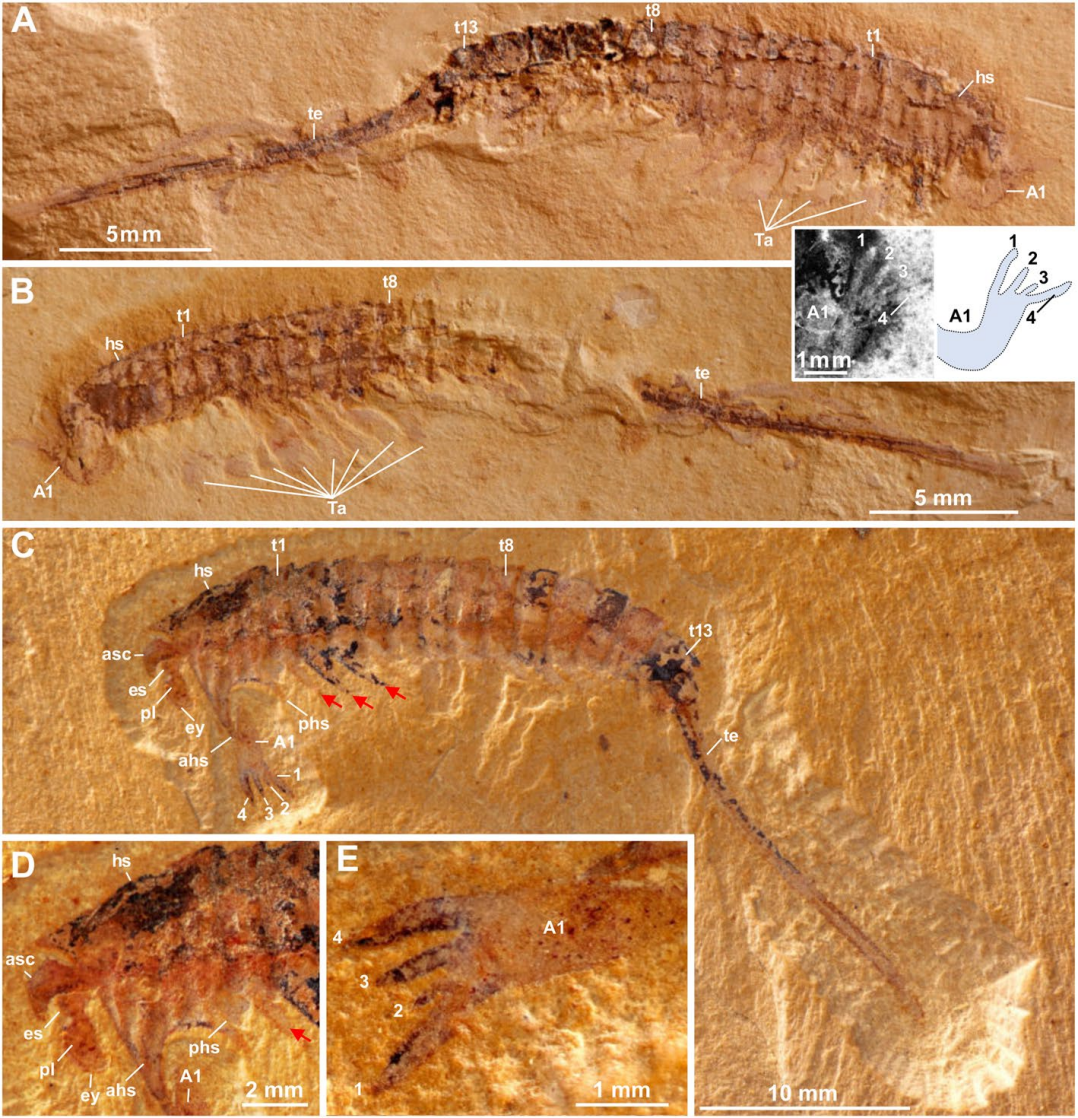
Macro-photographic images of previously published specimens of *Tanglangia longicaudata*. A Specimen Hz-f-7–228 (holotype, part). Inset: Fluorescent image and explanatory line drawing of the great appendage shown in **A**. **B** Specimen Hz-f-7–229 (holotype, counterpart). **C** Specimen YKLP 13917. **D**, **E** Close-ups of the head and great appendage shown in **C**, respectively

**Fig. 2:**
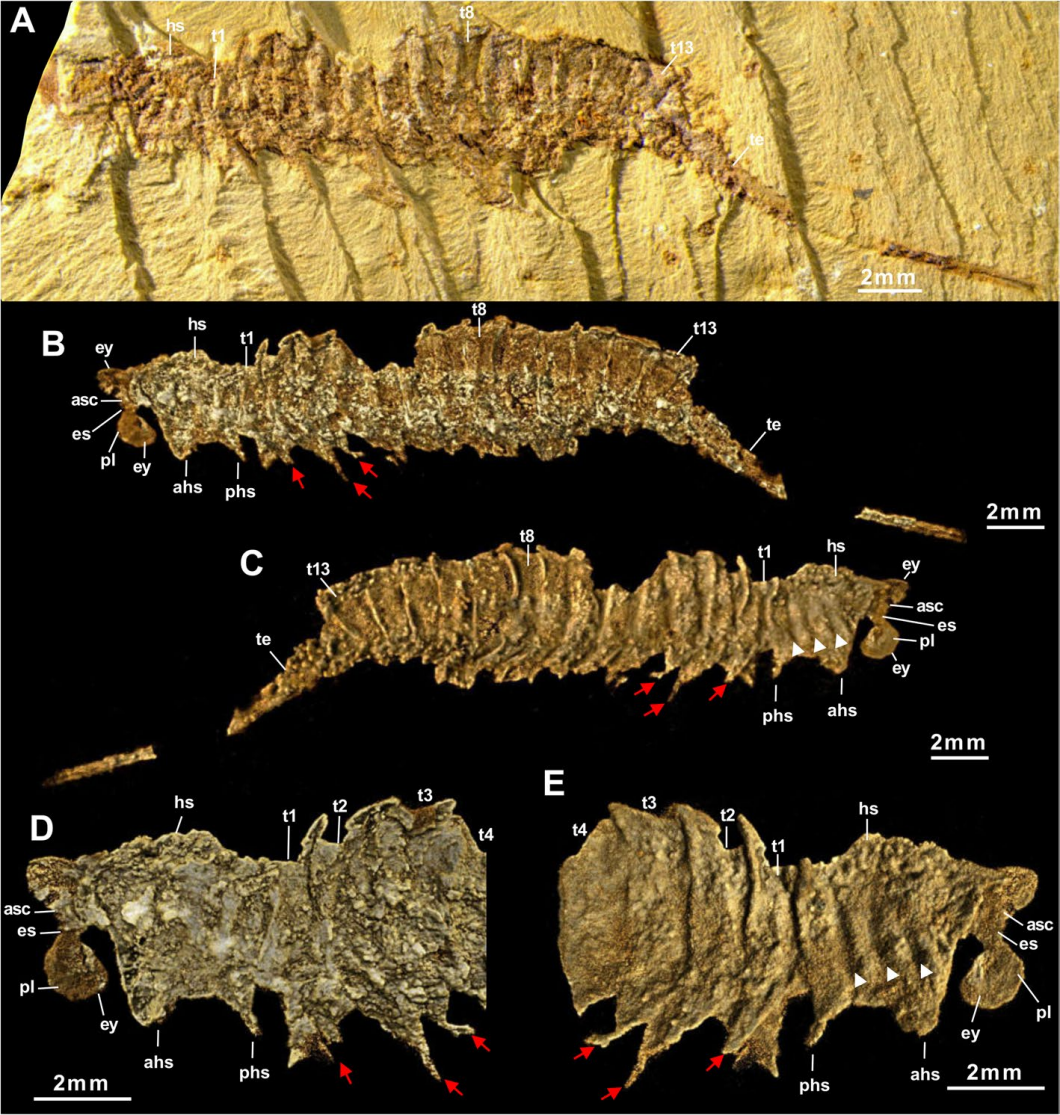
*Tanglangia longicaudata* specimen YKLP 17215a. **A** Macro-photographic image. **B**–**E** Drishti rendering results based on *μ*CT. **B** Same view as in **A**. **C** The other side of **B**. **D**, **E** Close-ups of anterior part of **B** and **C**, respectively

*Tanglangia longicaudata* was first described in 1999 by Luo and colleagues [17], p. 59, Plate 9, Fig. 2a, b; see also Fig. 1A, B], based on the following specimens—the holotype Hz-f-7–228 (part slab), Hz-f-7–229 (counterpart slab), and Hz-f-11–29. All specimens were collected from the Heilinpu Formation, Ercai village, Haikou town, Kunming area, Southwest China. According to the original description, this animal has a body (up to 35 mm) consisting of a head with a pair of great appendages bearing four pointed elements, a trunk with 12 segments each carrying a pair of “leaf-like” appendages, and an elongate telson. The head shield bears three furrows laterally, presumably indicating the presence of three head appendages. Without showing any evidence, the authors drew eyes on the head shield in their reconstruction. This short description was later slightly revised based on new material (Cat. No. NIGP 134797a, b) by changing the trunk segment number from 12 to 13, and by stating the telson being only the elongate and sharp spine itself, excluding the last trunk segment [18]. Moreover, Xu (2004) ([ref. [18]) also mentioned the possible presence of a pair of stalked eyes based on some sub-circular dark structures close to the head. No further studies on the morphology of *T. longicaudata* have been carried out ever since.

More recently, a new species of the same genus was described—that is, *Tanglangia rangatanga*. It was analyzed and described by Paterson and colleagues, uncovered from the lower Cambrian Emu Bay Shale, Australia [19], expanding the area and environment in which members of *Tanglangia* have occurred. *Tanglangia rangatanga* resembles *T. longicaudata* in several aspects, especially the general tergite organization and the remarkable telson. Due to the limited number of specimens and the preservation of the fossils, many details such as the proximal elements of the great appendages, the number of head appendages, and the morphology of the trunk appendages still remain a conundrum for this group of great appendage euarthropods.

Here, we combined the micro-computed tomography (*μ*CT) and computer-based 3D rendering techniques to investigate eight fossil specimens (including six individuals) of *Tanglangia longicaudata*, among which one specimen was from the original description back in 1999. We uncovered new ventral and appendicular morphological details of this enigmatic and yet understudied Chengjiang euarthropod. Additionally, we provide a comprehensive review of all specimens of *T. longicaudata* mentioned and depicted in the literature ever since, to disentangle its troublesome research history.

## Results

### Systematic palaeontology

Arthropoda Gravenhorst, 1843 ([20], see [21]).

Euarthropoda Lankester, 1904 [22].

“Megacheira” Hou et Bergström, 1997 [9].

[Yohoiida Simonetta et Delle Cave, 1975] [23].

[Yohoiidae Henriksen, 1928] [24].

*Tanglangia* Luo et Hu in Luo et al., 1999 [17].

*Type species. Tanglangia longicaudata* Luo et Hu in Luo et al., 1999 [17].

#### *Tanglangia longicaudata*

1999. *Tanglangia longicaudata* gen. et sp. nov. Luo et Hu, 1999 in Luo et al., pl 9, figs. 2 and 3 [17].

**Fig. 3:**
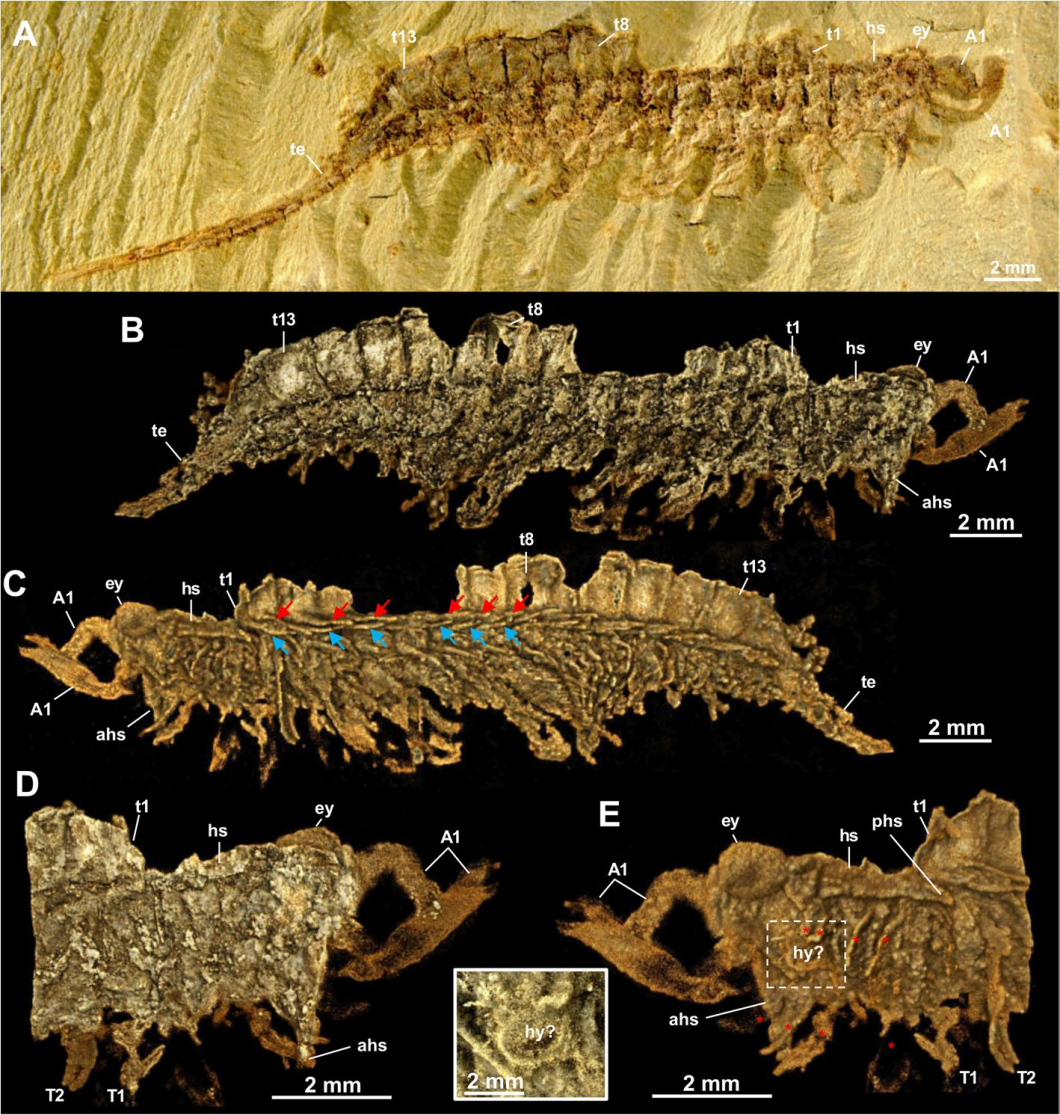
*Tanglangia longicaudata* specimen YKLP 17215b. **A** Macro-photographic image. **B**–**E** Drishti rendering results based on *μ*CT. **B** Same view as in **A**. **C** The other side of **B**. **D**, **E** Close-ups of anterior part of **B** and **C**, respectively. Inset: close-up of putative location of hypostome with marginal setae

2002. *Tanglangia longicaudata* Luo et Hu, 1999; Chen et al., pl 7, Fig. 3, pl 8, Fig. 2 [25].

**Fig. 4:**
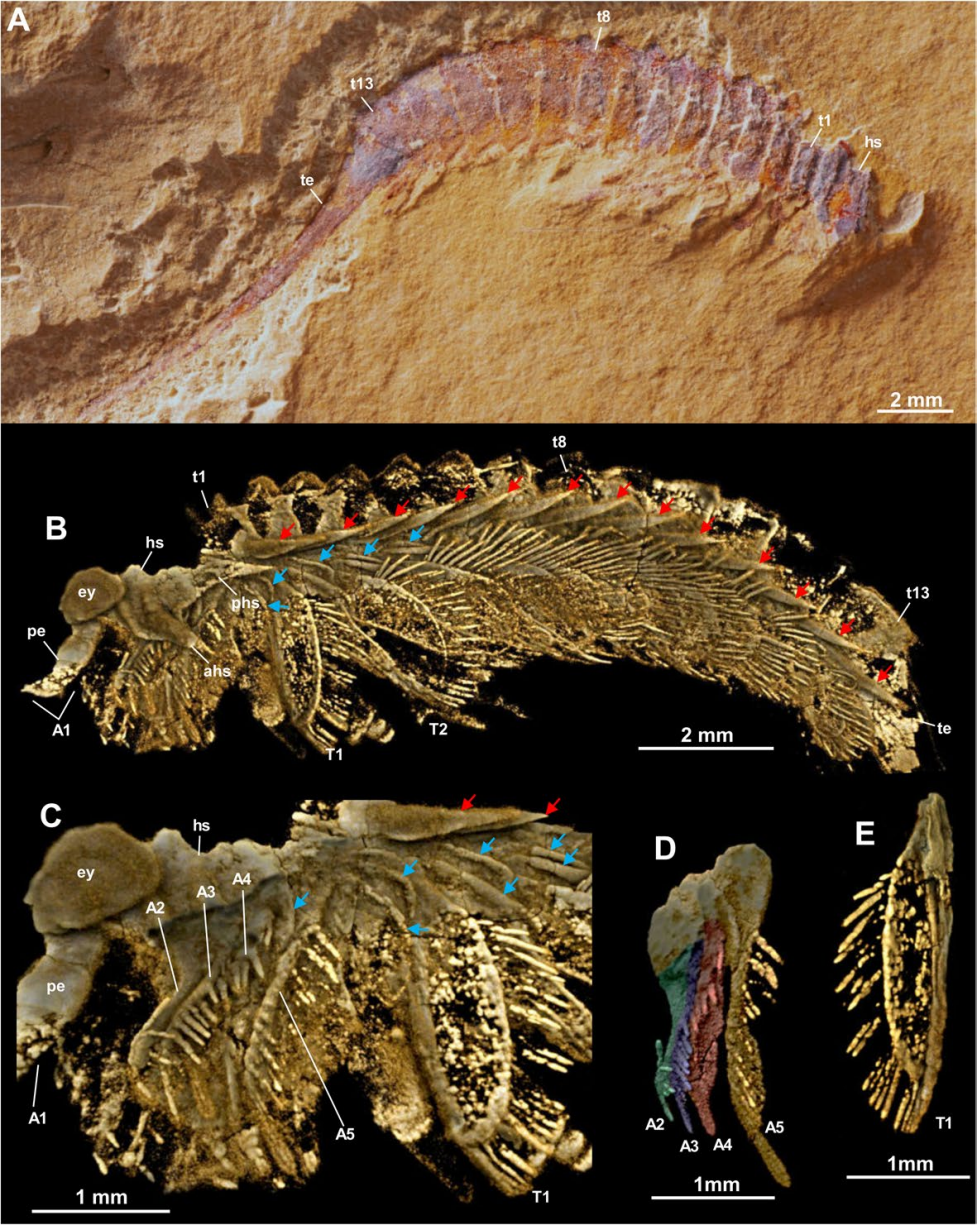
*Tanglangia longicaudata* specimen YKLP 17219. **A** Macro-photographic image. **B**–**E** Drishti rendering results based on *μ*CT. **B** The other side of **A**. **C** Close-up of the anterior part of **B** with partly removed anterior and posterior head spine. **D**, **E** Close-ups of isolated head appendages (A2–A5) and the first trunk appendage (T1) shown in **B** and **C**

2004. *Tanglangia longicaudata* [sic] Luo et Hu, 1999; Chen et al., Fig. 4D [16].

2004. *Tanglangia longicaudata* Luo et Hu, 1999; Chen, figs. 475–477. [26].

2004. *Tanglangia longicaudata* Luo et Hu, 1999; Xu, pl 1, figs. 1 and 2 [18].

2007. *Tanglangia longicaudata* [sic] Luo et Hu, 1999; Chen et al., Fig. 10C [27].

2012. *Tanglangia caudata* [sic] Luo et Hu, 1999; Schoenemann et Clarkson, pl 2, figs. c1–c4 [28].

2017. *Tanglangia caudata* Luo et Hu, 1999; Hou et al., figs. 20.18a–c (cf. Hou et al., 2004, figs. 16 and 20a–c) [29].

*Type material*. Holotype: Hz-f-7–228 (part) and Hz-f-7–229 (counterpart) in ref. ([17], Fig. 2a and b]).

*Type locality*. Yu’anshan Member of the Chiungchussu Formation, Dazi section of Haikou in Yunnan Province, South China.

*Emended diagnosis.* Body length ranges from 23.64 to 34.91 mm. Body becomes slightly curled at the posterior part and can be divided into head, trunk, and telson. Head shield bears three oblique grooves laterally. A pair of stalked eyes is located at the front of the ventral side of the head. A pair of great appendages each bearing four spines/fingers is located right behind the eyes. A hypostome with marginal setae is located behind the great appendages and adjacent to the first two pairs of biramous head appendages. Trunk consists of 13 segments, each bearing a pair of biramous appendages, ventrally. Endopodite of each biramous appendage is slender, number of elements uncertain, about seven to ten approximately, compared to other Megacheira. Exopodites are leaf-shaped or flap-like, with long marginal setae. Telson is as long as the trunk, with a robust proximal portion, gradually becoming slender and terminating in a sharp spine. Modified after ref. [17] and ref. [18].

*Material.* YKLP 13917, YKLP 17215a, YKLP 17215b, YKLP 17217, YKLP 17218, YKLP 17219, Hz-f-7–228, Hz-f-7–229.

### Description

#### Body

**Fig. 5:**
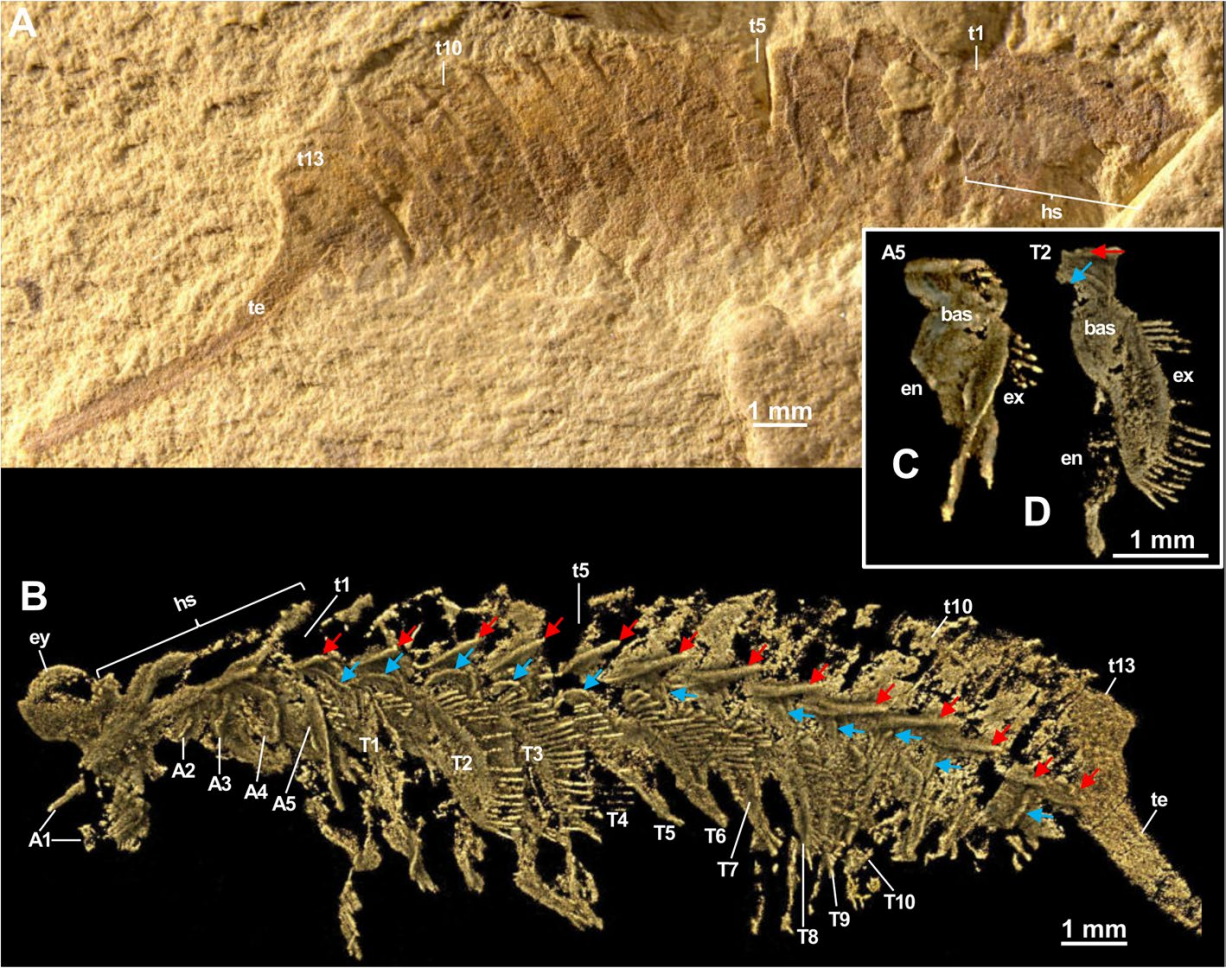
*Tanglangia longicaudata* specimen YKLP 17217. **A** Macro-photographic image. **B**–**D** Drishti rendering results based on *μ*CT. **B** The other side of **A**. **C**, **D** Rotated close-ups of isolated appendages A5 and T2 shown in **B**

Body of *Tanglangia longicaudata* consists of the head, the trunk, and the telson and becomes slightly curled at the posterior part (Fig. 1A, B, Additional file 1: Fig. S1). Due to the preservation, measurements of the full-body length from the anterior-most point of the head shield to the tip of the telson are only available for the following specimens: Hz-f-7–229, 34.91 mm (Fig. 1B, Additional file 1: Fig. S1B); YKLP 17215, 33.58 mm (Figs. 2 and 3), and YKLP 17217, 23.64 mm (Fig. 5, Additional file 1: Fig. S2B).

#### Head organization

Head of *Tanglangia longicaudata* has a dorsal shield with three intersegmental furrows indicating the segmentation on the ventral side of the head (Figs. 1C, D and 2C, E). The head shield covers six segments underneath. From anterior to posterior, these are the ocular segment carrying a pair of stalked eyes (also including the anterior sclerite), the segment bearing a pair of great appendages, and four segments each bearing a pair of biramous appendages (Figs. 2, 3E, 4C, D, and 5B). A rounded structure, possibly presenting the hypostome is identified behind the great appendages on YKLP 17215b (Fig. 3E, inset).

#### Eyes

**Fig. 6:**
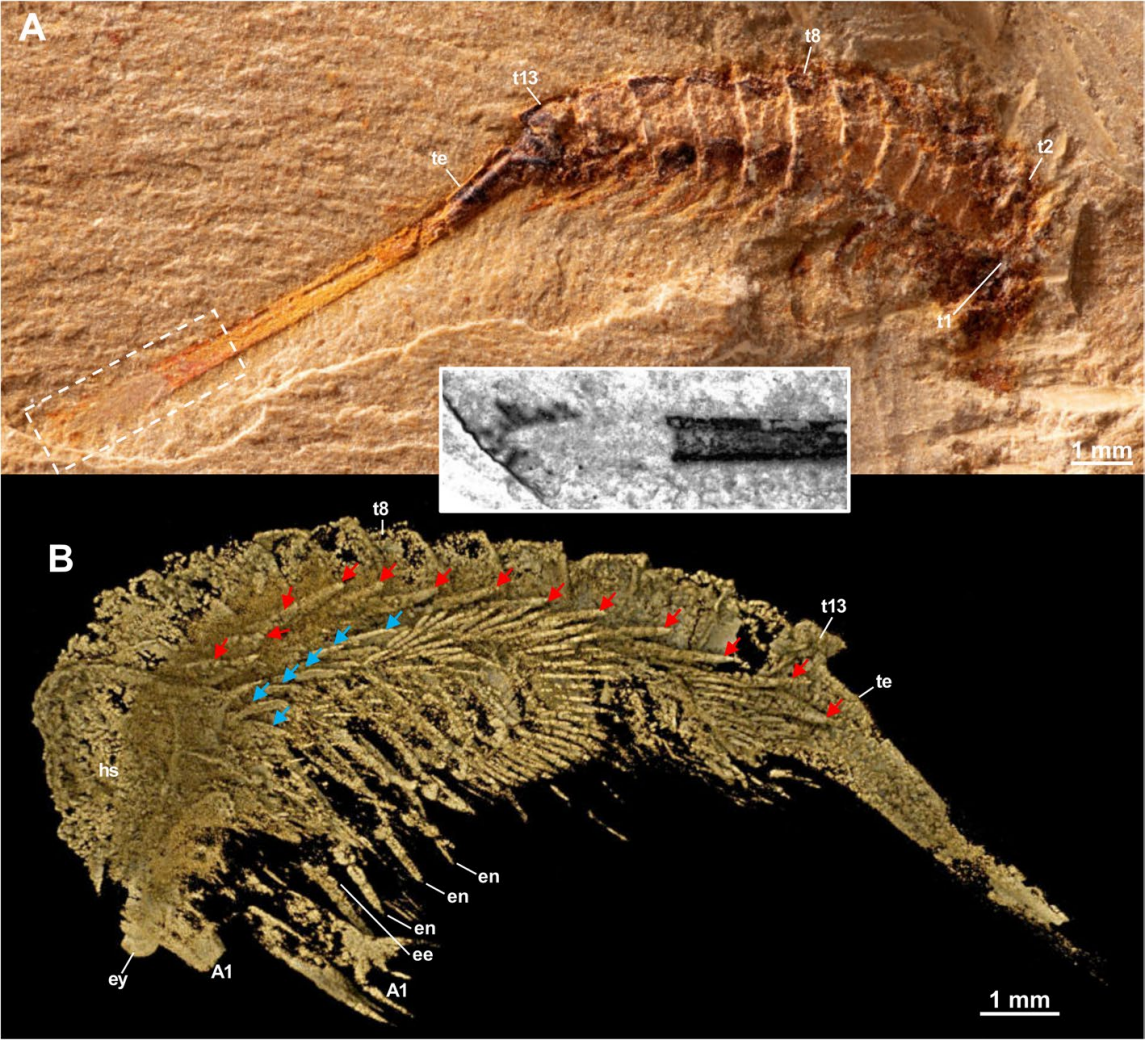
*Tanglangia longicaudata* specimen YKLP 17218. **A** Macro-photographic image. Inset: fluorescent image showing the terminal end of the telson shown in **A**. **B** Drishti rendering result based on *μ*CT, the other side of **A**

**Fig. 7:**
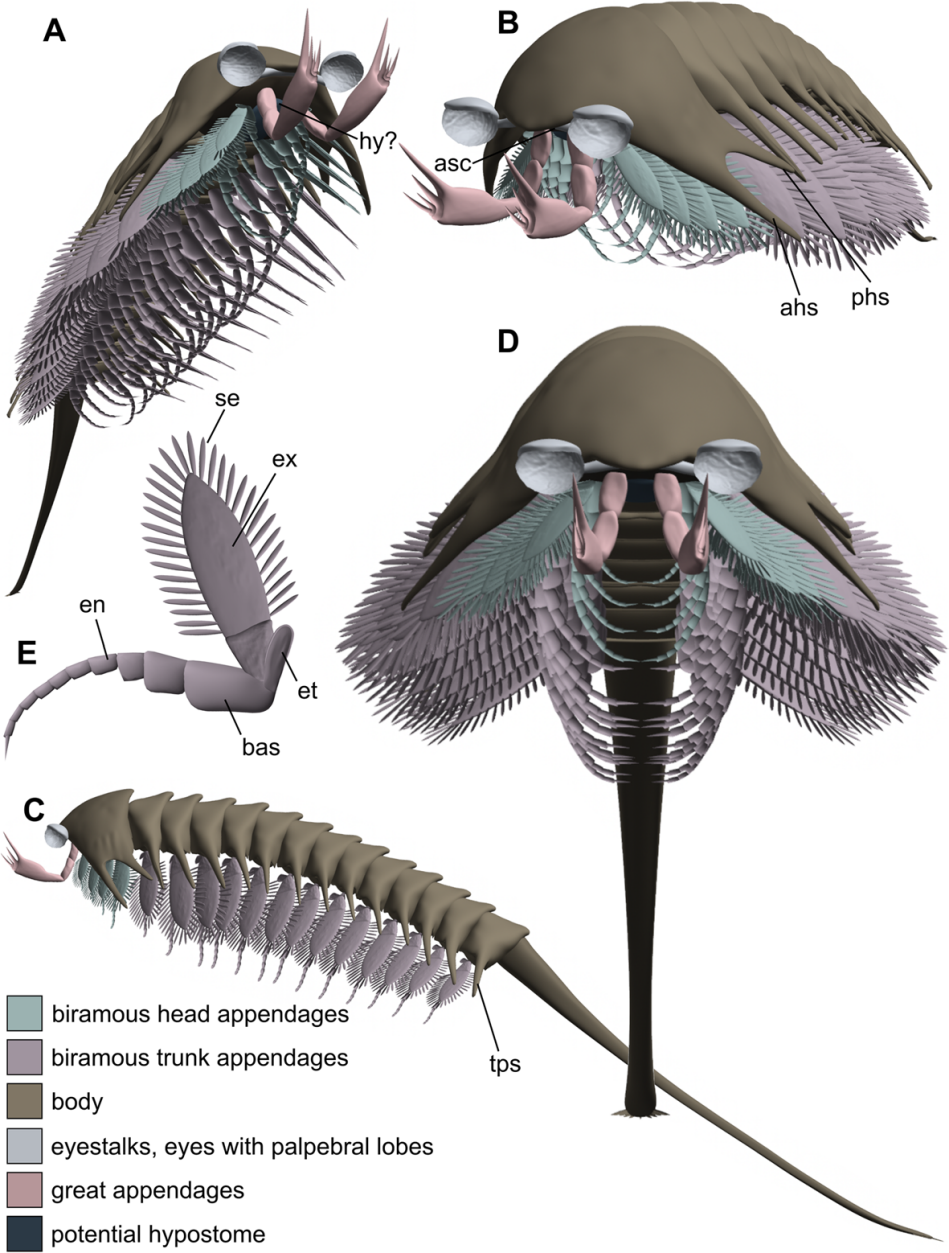
Three-dimensional Blender model of *Tanglangia longicaudata*. **A** Antero-ventral oblique view. **B** Antero-lateral oblique view. **C** Lateral left view. **D** Frontal view. **E** Biramous trunk appendage. Not to scale

Eyes are preserved on most of the studied specimens: YKLP 13917 (Fig. 1C, D), YKLP 17215a (Fig. 2), YKLP 17215b (Fig. 3), YKLP 17219 (Fig. 4B, C), YKLP 17217 (Fig. 5B), YKLP 17218 (Fig. 6B). They occur as a pair of cup-shaped structures protruding from the anterior-most component (anterior sclerite) and are covered by the anterior portion of the head shield. Each eye is dorsally covered by a so-called palpebral lobe (Figs. 1C, D, 2B–E, and 7, see ref. [28]). The stalks do exhibit at least two joints (Fig. 1D). YKLP 17215a preserves one eye and stalk, visible only in tomographic, but not in macro-photographic images (Fig. 2). The stalk measures around 0.4 mm in length and the “eyeball” around 1.1 mm in diameter. YKLP 17215b preserves one eye with the “eyeball” being visible on both tomographic and macro-photographic images measuring 1.1 mm in diameter (Fig. 3). The eyestalk is not preserved. YKLP 17219 preserves one eye with around 1.1 mm in diameter, with its stalk heavily compressed during fossilization (Fig. 4B, C). YKLP 17217 preserves one eye of around 1.2 mm in diameter, without a stalk (Fig. 5B, Additional file 1: Fig. S2B). YKLP 17218 preserves one eye of around 0.91 mm in diameter, with a stalk (Fig. 6B, Additional file 1: Fig. S2C).

#### Great appendages

A pair of great appendages (GAs) are found not only on the holotype (Fig. 1A, B, inset), but also on several other specimens (Figs. 1C, E, 3, 4B, C, and 5B). Among these, only YKLP 17215b preserves the GAs nearly in its full length, though element borders are not clearly visible (Fig. 3). The left GA is curved, and only partly preserved with the peduncle elements (no borders visible) and parts of the claw elements. The right GA is better preserved but lacks parts of the distal-most claw elements. It measures ca. 3.8 mm in length and about 0.5 mm in overall width.

The entire GA in *T. longicaudata* is composed of six elements, with a robust peduncle comprised of two elements (best visible in specimen YKLP 17219, Fig. 4B, C with around 1.56 mm in length and 0.78 mm width at the thickest region), followed by the first robust claw element and presumably three slender elements, ending in fine spines. The first claw element is long and slightly curved forward alongside its dorsal margin; it is wide and thickened most in the middle part (Fig. 3B–E). Second to forth claw elements are not well-pyritized in specimen YKLP 17215b and thus not clearly visible in the *μ*CT data (Fig. 3), but can be clearly observed in the herein presented photographs of the holotype part Hz-f-7–228 (Fig. 1A, inset) and in specimen YKLP 13917 (Fig. 1C, E). In Hz-f-7–228, claw element 3 seems to be the smallest, while in YKLP 13917 claw element 2 appears to be the smallest. In the latter specimen, the clearly visible great appendage furthermore might be disjointed from the body and also flipped.

Nonetheless, the overall shape of the preserved GA parts in the *T. longicaudata* specimens suggest a similar morphology like in *J. multisegmentalis*, though in this specimen only one peduncle element was found ([14], Fig. 6A]).

#### Biramous head appendages

In the head of *T. longicaudata*, the GAs are followed by several pairs of biramous appendages. Based on the exoskeletal furrows on the dorsal side of the head shield, Luo and colleagues suggested three pairs of biramous head appendages in the original description of the animal ([17] p. 59]). Despite the furrows that are also observed in this study (Fig. 2C, E), we identified four pairs of biramous appendages in the head of *T. longicaudata* from three specimens (Figs. 3E, 4C, D, and 5B). By digitally removing the lateral margin of the head shield of YKLP 17219, we are able to show that each biramous head appendage bears an exopodite fringed with marginal setae (Fig. 4C, D). A 3D model of YKLP 17217 suggests a leaf-shape for the exopodite of each biramous head appendage (Fig. 5B), while multi-segmented endopodite of those appendages can be seen in the 3D model of YKLP 17218 (Fig. 6B, Additional file 1: Fig. S2C), suggesting a similar morphology shared by the biramous appendages in the head and trunk (e.g., Fig. 5C, D).

#### Trunk

The trunk of *T. longicaudata* consists of 13 segments each covered by a tergite dorsally and carrying one pair of biramous appendages ventrally. The trunk measures approximately 17.62 mm in length for specimen YKLP 17215a, 15.48 mm in YKLP 17215b, 11.90 mm in YKLP 17219, 15.77 mm in YKLP 17217, 7.36 mm in YKLP 17218, and 16.32 mm for specimen Hz-f-7–229. Tergite length on average is between 1.34 mm in YKLP 17215a, 1.14 mm in YKLP 17215b, 0.9 mm in YKLP 17219, 0.9 mm in YKLP 17217, 0.71 mm in YKLP 17218, and 1.25 mm in Hz-f-7–229. Length of the tergites is nearly the same, with the 13^th^ tergite being slightly longer than the others.

Every trunk tergite exhibits a pair of spiky tergopleurae (Figs. 1C, 2B–E, 3C, 4B, C, 5B, and 6B). On the laterally compressed specimens, they are all imbricate and folded posteriorly. On specimens YKLP 17219 (Fig. 4) and YKLP 17217 (Fig. 5), the tergopleurae increase in size from the anterior to the middle region of the body, and then decrease gradually towards the posterior end.

#### Trunk appendages

Each of the 13 trunk segments bears one pair of biramous appendages that share a similar morphology with those in the head (e.g., Fig. 5B). The endopodite is slightly wider at the base, gradually narrowing distally, and tapered at the end, and may be composed of seven to ten elements (Fig. 6B, Additional file 1: Fig. S2C). YKLP 17217 shows that the endopodites become longer from the first (2.1 mm) to the third (2.8 mm) trunk appendage, while those of the fourth and ninth trunk appendages measure about 3.1 mm. The endopodites of the more posterior trunk appendages gradually become shorter, from 2.9 to 1.2 mm (Fig. 5B). The exopodite of all biramous appendages consists of a rounded triangular proximal part and an oval distal part with marginal setae (Fig. 5B–D). Specimens of YKLP 17219 (Fig. 4), YKLP 17217 (Fig. 5), and YKLP 17218 (Fig. 6) indicate that the average length of the exopodites of the fourth to ninth trunk appendages is approximately 1.9 mm and is shorter than their respective endopodites. From anterior to posterior, there is an increase in length for the exopodites of the first three pairs of trunk appendages, and a decrease for the last four pairs. Number of the marginal setae on the exopodites varies from 15 to slightly more than 20, with a size range of size between 0.3 and 0.6 mm. On several specimens, one to two small flap-like structures with a thickened margin are observed near the most proximal part of the trunk appendages, forming a line below the line of tergopleural spines (Figs. 3C, 4B, C, 5B, and 6B). Given the position and the size of those flap-like structures, we interpret them as putative exites that have been found in other megacheirans (i.e., *Leanchoilia illecebrosa* and *L. obesa*) and artiopods (i.e., *Naraoia spinosa* and *Retifacies abnormalis*) from Chengjiang (see ref. [30]).

#### Telson

The 13^th^ trunk tergite is followed by a straight and elongated telson that is as long as the head plus trunk—the most distinguishable feature of *T. longicaudata* (Figs. 1, 2, 3, 4 and 6). The telson is terminated into a narrow dagger-shaped end fringed with several short marginal setae (Fig. 6A, inset).

### Three-dimensional reconstruction

Our three-dimensional model (Fig. 7) represents the updated morphology of *Tanglangia longicaudata*, based on evidence from advanced *μ*CT-technique on the one hand, and a comparative review of the literature on the other. We present a specimen showing a modest curved body with endopodites slightly bent ventrally and laterally splayed exopodites.

## Discussion

### *Tanglangia longicaudata* and other great appendage euarthropods within the taxon Megacheira

We have presented, for the first time, a detailed examination of the ventral and appendicular morphology of *Tanglangia longicaudata*, an early Cambrian Chengjiang euarthropod from China. In its initial characterization in 1999 [17], the phylogenetic placement of this species was not firmly established. Subsequently, Xu in 2004 ([18], p. 329]) was the pioneer in suggesting potential affiliations for *T. longicaudata*, classifying it within the family Yohoiidae Henriksen, 1928, nested within the broader group Megacheira.

The taxonomic identification of the Megacheira, particularly the great appendage euarthropods, remains a subject of intense debate. The attribution of the term “great appendages” to either the protocerebrum [31], deutocerebrum [10, 13, 32], or tritocerebrum [33] has been extensively deliberated. Furthermore, the broader categorization of the Megacheira as either belonging to Chelicerata [10, 11, 13, 15, 16, 34, 35] or representing stem-group euarthropods [33, 36–39] remains a topic of significant contention.

The chelicerae found in contemporary chelicerates exhibit a deutocerebral identity, as determined by hox gene expression domains [40]. Therefore, if the innervation of the “great appendages” is also deutocerebral, as proposed by ref. [13], these structures within Megacheira would be considered homologous to the chelicerae in chelicerates, thus classifying them as genuine stem-group chelicerates.

Nevertheless, the debate regarding the monophyletic or paraphyletic nature of Megacheira has been explored [41, 42]. This debate is particularly relevant, given that one member, *Fortiforceps foliosa*, is suggested to have possessed antennae [9], which would be inconsistent with the presumed ancestral pattern of chelicerates.

Due to the limited availability of material and consequent lack of detailed morphological information, *T. longicaudata* has been infrequently considered in studies addressing phylogenetic relationships among early Cambrian euarthropods and within Megacheira ([16], Fig. 6; [43], extended data figs. 9 and 10]).

Nevertheless, in both studies, it was identified as a sister taxon to *Yohoia tenuis*, with both taxa forming a sister group to the clade composed of *Jianfengia multisegmentalis* and *F. foliosa*, all nested within Megacheira.

**Fig. 8:**
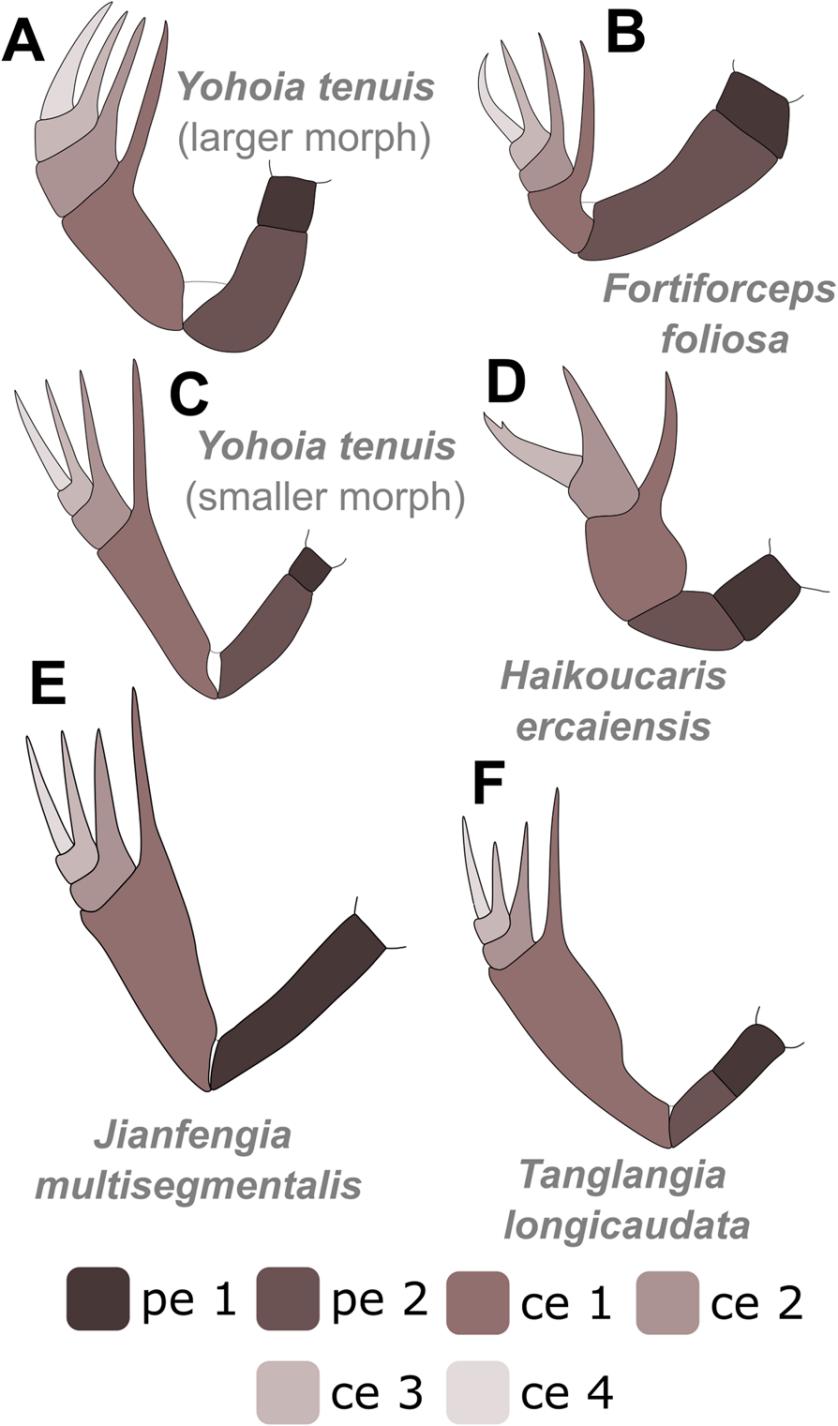
Comparison of the raptorial great appendages among certain Megacheira. Redrawn after: **A**–**D** ref. [15]. **E** ref. [14]. **F** This study. Note that regarding literature, *J. multisegmentalis* is the only described species to solely exhibit one peduncle element. Not to scale

Indeed, these four taxa (*T. longicaudata*, *Y. tenuis*, *J. multisegmentalis*, *F. foliosa*) exhibit numerous morphological similarities. Each possesses a pair of great appendages (GAs), which are relatively short and lack filamentous distal parts compared to *Leanchoilia* sp. or *Alalcomenaeus* sp. Additionally, they all feature a pair of stalked eyes, lack antennae (although *F. foliosa*’s antennal presence is debatable, as mentioned in ref. ([9], p. 37]), and share a set of similarly shaped biramous trunk appendages, as well as an unsegmented telson. Concerning post-GA head appendages, the situation is less clear. Some megacheirans exhibited four post-GA head appendages: *Haikoucaris ercaiensis* [14], Fig. 8B; ([16], Fig. 8A, B]), *Leanchoilia illecebrosa* [44], Fig. 1G, H], *L. superlata* [10], and *J. multisegmentalis* ([14], figs. 1, 2, 3, 4, 5, 6 and 7]). Conversely, others were found to bear only three, such as *Yohoia tenuis* ([15], p. 384]) and earlier studies on *T. longicaudata* ([17], p. 59; [18], p. 329]), which was similarly speculated for the Australian counterpart *T. rangatanga* ([19], Figs. 3C, D and 4B–D]). However, the presence of four post-GA head appendages aligns with the current understanding of arthropod head evolution—a six-segmented head (ocular segment carrying the eyes, post-ocular segment involving the GA, four subsequent biramous appendages bearing segments) may represent the ancestral condition ([14], p. 9]). For three of our examined specimens (YKLP 17215, Fig. 3E; YKLP 17219, Fig. 4C, D; YKLP 17217, Fig. 5B), we were able to demonstrate the presence of four post-GA head appendages.

The great appendages of Megacheira (Fig. 8) have been previously construed to consist of either six elements (*Y. tenuis*, *J. multisegmentalis*, *F. foliosa*) or five (*H. ercaiensis*), where the initial two elements consistently form the peduncle, and the subsequent ones pertain to the claw elements ([15], fig. 11; [16], Fig. 5]).

The GAs in *J. multisegmentalis* were described as resembling the slender morphology of the 2^nd^ peduncle element and 1^st^ claw element found in the small morphotype of *Y. tenuis*. Conversely, the GAs in *F. foliosa* were noted to resemble the larger morphotype of *Y. tenuis*, with a relatively stout 2^nd^ peduncle element and a less elongated 1^st^ claw element [15], p. 384].

However, Zhang et al. (2022) ([ref. [14]), Fig. 7] proposed an alternative interpretation of the GAs in *J. multisegmentalis*, suggesting five elements without a differentiation of the peduncle into two separate elements.

Our analysis of *T. longicaudata* unveiled six elements, featuring a bipartite peduncle and four elements bearing spines. This indicates one additional peduncle element compared to the original description ([17], p. 59]). Ultimately, the determination of whether the 3^rd^ claw element (as in Hz-f-7–228) or the 2^nd^ (as in YKLP 13917) is shorter cannot be asserted with certainty; such differences may stem from preservation biases or compaction effects.

The number of trunk segments varies among taxa, with *J. multisegmentalis* having 22, *F. foliosa* 20, and *Y. tenuis*, *H. ercaiensis*, and *T. longicaudata* possessing 13 trunk segments each. Notably, *T. longicaudata* is distinctive for its elongated telson. In *Y. tenuis*, the telson is short and paddle-shaped ([15], p. 384]), while in *J. multisegmentalis*, it manifests as a short, rod-like structure terminating in a pointed end ([29], p. 182]). Conversely, in *F. foliosa*, the telson exhibits a fan-like morphology, adorned with fine bristles at the edges and comprises a median region flanked by a pair of elongated lateral blades ([29], p. 182]).

### The Australian Tanglangia rangatanga

In 2015, *T. rangatanga*, a new species, was documented from the Emu Bay Shale Konservat-Lagerstätte on Kangaroo Island, South Australia, thereby expanding the geographic and stratigraphic range of the genus [19]. To date, this represents the sole additional *Tanglangia* species recognized and described alongside *T. longicaudata*. Its general morphology closely resembles the Chinese species, including the number of tergites, the elongated telson, and the presence of tergopleural spines ([19], figs. 1, and 2]). However, numerous morphological features, such as the great appendages (GAs), eyes, and appendage details, remain unobservable due to incomplete preservation of the material.

A notable distinction lies in the presence of teardrop-shaped nodi on the lateral side of the tergites (which ref. [19] refers to as tergopleurae) in *T. rangatanga*, an attribute entirely absent in *T. longicaudata*. Additionally, *T. rangatanga* lacks the two head spines observed in some literature specimens of *T. longicaudata*, a discrepancy that may be attributed to the scarcity of well-preserved head material. Among the available specimens, only one exhibited the posterior head portion, albeit lacking a spine at the postero-lateral corner ([19], Fig. 2]). Unlike *T. longicaudata*, *T. rangatanga* was interpreted to bear only three post-GA head appendages, which the authors inferred from the intersegmental furrows. It is likely that the Australian *T. rangatanga* also exhibited four post-GA head appendages.

The description of the Australian *Tanglangia* species relies on the holotype, eight paratypes, and nine additional unfigured specimens. Unfortunately, the type of fossilization makes them unsuitable for in-depth *μ*CT research. Consequently, the genuine nature of the ventral morphology in *T. rangatanga* might remain elusive, until additional specimens might be discovered preserving appendicular structures.

### Autecology of Tanglangia longicaudata

Considering the prominent eyes, the flexible trunk with the flap-like, leaf-shaped exopodites, the elongated telson, and the presumably predatory “great appendages,” we posit *T. longicaudata* as an active predator and adept swimmer.

In 2012, Schoenemann and Clarkson [28] conducted an analysis of the eyes in various Chengjiang arthropods based on the amount of freely available light. Their findings regarding the design of *T. longicaudata*’s large eyes and the calculated parameters (eye parameter ~ 38 μm rad) suggests that those eyes worked effectively under low-light conditions. This could potentially hint on an adaptation to “deeper” waters or turbid waters with a lot of sediment, or further to a crepuscular or nocturnal lifestyle. Chen in 2004 ([26], p. 15]) proposed that the Chengjiang environment may have had depths of around 70–120 m. Consequently, *T. longicaudata* may have occupied a niche in “deeper” sea regions, aligning with the adaptations of its visual system. Moreover, just recently, the Chengjiang biota was found to once living in a deltaic environment characterized by unstable salinity conditions and frequent storm-floods, resulting in a high sedimentation rate [45]. Despite these conditions, the waters likely remained oxygen and nutrient-rich.

The flexibility of the trunk is evidenced in specimen Hz-f-11–29 [17], pl. 8, Fig. 3; also refigured in ref. [25], pl. 8, Fig. 2, oriented 180° towards the image in ref. [17], as well as in specimen NIGP 134797a, b [18], pl. 1, figs. 1 and 2]. These fossils exhibit a degree of trunk flexibility not observed in other *T. longicaudata* specimens, which tend to preserve a more elongated body form. A slight degree of trunk flexibility in the Burgess Shale taxon *Y. tenuis* was similarly demonstrated yet ([15], Fig. 3B–D, F]).

The possession of a flexible trunk conferred increased agility to the organism during foraging activities, while the elongated telson in *T. longicaudata*, reaching lengths comparable to the entire body in some specimens, likely played a role in stabilization and maneuvering.

Concerning its prey, we propose that *T. longicaudata* primarily targeted soft-bodied organisms. Although a comprehensive morpho-functional and kinematic analysis of the great appendages (GAs) in megacheirans is outstanding, a recent study integrating finite element analyses, computational fluid dynamics, and range of motion analyses suggested that *Anomalocaris canadensis*, a member of Radiodonta (the larger “great appendage” arthropods), also likely fed rather on soft prey. The investigation demonstrated potential damage to the great appendages when employed in hunting hard-shelled groups such as trilobites [6].

Potential food sources for great appendage arthropods have been previously proposed for *Y. tenuis*, which was speculated potentially feeding on small-sized taxa like bradoriids ([15], fig. 13]). While *Y. tenuis* is a Burgess Shale arthropod, bradoriids are also present in the Chengjiang biota, exemplified by species like *Kunmingella douvillei* or *Kuanyangella cheni* [46].

Ultimately, morpho-functional studies employing kinematic models of the great appendages in specific arthropods with such structures could elucidate hunting modes and offer insights into potential prey items.

### Tanglangia longicaudata—critical literature remarks

To date, only eight individual specimens of *T. longicaudata* have been documented through photographs, disseminated across various publications and books spanning from 1999 to 2017. Unfortunately, the historical record of these specimens has encountered challenges, including refiguring, inaccurate assignment, and mirrored or upside-down images, contributing to confusion (Table 1).

**Table 1:** Overview of all available specimens of *Tanglangia longicaudata* yet depicted in literature since 1999, as well as in this study

Coll. No	Figure	Reference
Hz-f-7–228	Figure 1A Fig. S1A	**-this study** **-original:** Luo et al. (1999; pl. 9, Fig. 2a) [17] **-also depicted in:** -Chen et al. (2002; pl. 7, Fig. 3b) [25] -Chen (2004; fig. 476, lower image) [26]
Hz-f-7–229	Figure 1B Fig. S1B	**-this study** **-original**: Luo et al. (1999; pl. 9, Fig. 2b) [17] **-also depicted in:** -Chen et al. (2002; pl. 7, Fig. 3a) [25] -Chen (2004; fig. 476, upper image) [26] -Chen et al. (2004; Fig. 4D) [16] -Chen et al. (2007; fig. 10C) [27]
YKLP 13917	Figure 1C–E	**-this study** **-original**: Hou et al. (2017; fig. 20.18a) [29]
YKLP 17215a	Figure 2	**-this study**
YKLP 17215b	Figure 3	**-this study**
YKLP 17219	Figure 4 Fig. S2A	**-this study**
YKLP 17217	Figure 5 Fig. S2B Fig. S3	**-this study**
YKLP 17218	Figure 6 Fig. S2C	**-this study**
Hz-f-11–29		**-original**: Luo et al. (1999; pl. 9, Fig. 3, upside down) [17] **-also depicted in:** -Chen et al. (2002; pl. 8, Fig. 2) [25]
?477		**-original**: Chen (2004; fig. 477) [26]
NIGP 134797a,b		**-original**: Xu (2004; pl. 1, Figs. 1 and 2) [18]
?2c1-c4		**-original**: Schoenemann & Clarkson (2012; pl. 2, figs. 2c1-c4) [28]
YKLP 13918		**-original**: Hou et al. (2017; fig. 20.18b) [29]
RCCBYU 10268		**-original**: Hou et al. (2017; fig. 20.18c) [29]

The initial description of *T. longicaudata* occurred in 1999 by Luo and Hou [17]. The authors derived their depiction and illustration (“illustration 15”, p. 59) from three specimens: the designated holotype featuring the part Hz-f-7–228 and the counterpart Hz-f-7–229, along with specimen Hz-f-11–29, displaying morphological differences upon initial examination.

However, the same specimen Hz-f-11–29 was subsequently refigured in ref. [25]. In this compilation, the photograph of the specimen is not only colored (in contrast to the black and white images in the original description in ref. [17]) but also “correctly” oriented, that is, rotated 180° in alignment with the image of the same specimen in ref. [17]. Chen and colleagues in 2002 [25] also refigured the holotype part Hz-f-7–228 and counterpart Hz-f-7–229. Upon closer inspection, it becomes evident that the authors mistakenly interchanged the collection numbers, with their assigned Hz-f-7–228 representing a photograph of specimen Hz-f-7–229, and vice versa. Aside from the confusion, it is not permissible to have two holotype specimens with distinct published collection numbers according to the International Code of Zoological Nomenclature (ICZN). The original description should have designated a single specimen number with a and b to categorize them as part and counterpart of the holotype, respectively.

Chen in 2004 [26] also presented illustrations of the holotype part Hz-f-7–228 and counterpart Hz-f-7–229 of *T. longicaudata*. Additionally, this publication introduced a fourth depicted specimen, denoted as specimen ?477 herein by us due to the absence of an assigned specimen number ([26], fig. 477]). Notably, the authors did not associate any collection number with this depicted specimen. However, it is worth noting that their fig. 476 indeed corresponds to Hz-f-7–228 and Hz-f-7–229. Two more specimens of *T. longicaudata* were also included, featured in fig. 478 and fig. 479 in ref. [26], respectively. Nevertheless, we have chosen not to categorize them as fossils representing *T. longicaudata* since they lacked description in ref. [26], and the limited morphological information given there suggests potential affiliation with *J. multisegmentalis* or *F. foliosa* rather. In the case of the specimen in fig. 478 in ref. [26], only the posterior portion is preserved, exhibiting the proximal part of the telson, a slender dark structure, and impressions of nine tergites (t13-t5). The anterior tergites (t4-t1) are challenging to discern clearly due to ambiguous boundaries. Notably, critical features of *T. longicaudata*, such as the head and primarily the telson, are not preserved. Conversely, the specimen in fig. 479 exclusively preserves the anterior part, bearing a closer resemblance to *J. multisegmentalis* (compare Fig. 1 in ref. [14]). In our interpretation, the morphological details provided for the specimens in figs. 478 and 479 in ref. [26] do not suffice for their confident assignment to the species *T. longicaudata*. Therefore, we assume that this publication depicted three rather than five specimens. Notably, these putative *T. longicaudata* specimens lacking proper labels were not subsequently refigured in any subsequent *T. longicaudata* studies.

Also in 2004, Chen and colleagues [16] introduced the first description of *H. ercaiensis*, identified as a close relative of *T. longicaudata*. Consequently, they refigured the well-known holotype counterpart Hz-f-7–229 of *T. longicaudata* from its original description, but inaccurately designating it as specimen number EC 15003. Additionally, upon closer examination, the fossil depicted in Fig. 4D in ref. [16] is identified as a mirrored photograph of the holotype part Hz-f-7–228, mislabeled as Hz-f-7–229. Chen and colleagues in 2007 [27] later acknowledged the mislabeling in ref. [16] as collection number EC 15003, yet they again portrayed Hz-f-7–229. Similarly, this study presented a mirrored photograph of specimen Hz-f-7–228, misidentified as Hz-f-7–229.

The fifth specimen (NIGP 134797a, b) of *T. longicaudata* was disclosed in ref. [18]. This morphological study, the first since 1999, contributed to the anatomical understanding of *T. longicaudata* by clarifying that the telson is represented solely by the “sword-shaped” tail, establishing that this species possesses 13 trunk segments.

The sixth specimen, featured in ref. [28], focused on the visual system of Cambrian euarthropods, including *T. longicaudata*. Regrettably, no collection numbers are available from this study; thus, we refer to this specimen as ?2c1-c4 (see Table 1).

The last three specimens (YKLP 13917, YKLP 13918, and RCCBYU 10268), exhibiting nearly complete preserved animals in total length, were illustrated in ref. ([29], fig. 20.18a–c]). Two of these specimens (YKLP 13917, YKLP 13918) were also incorporated in our re-examination.

In total, there are nine *T. longicaudata* fossil specimens yet portrayed and depicted in literature since; however, only eight represent individual animals, as Hz-f-7–228 and Hz-f-7–229 constitute the part and counterpart of the holotype, thus originating from the same once-living individual.

### Morphological variation within Tanglangia longicaudata

The morphological variations observed across all documented specimens of *T. longicaudata* in the literature from 1999 to 2017 are substantial, and the challenges associated with referenced specimen photographs have been highlighted previously.

Notably, certain morphological features are inconsistently present among specimens, with notable differences in the presence of anterior and posterior head spines. These features are prominently visible in all three specimens illustrated in ref. [29]. Specimen YKLP 13918 ([29], fig. 20.18b]) exclusively exhibits the anterior head spine. Furthermore, in YKLP 13917 (our Fig. 1C–E; ref. [29], fig. 20.18a), the spines are posteriorly oriented, whereas in RCCBYU 10268 ([29], fig. 20.18c), they face anteriorly. An additional specimen (?477 in ref. [26], fig. 277) appears to preserve primarily the anterior head spine.

In our investigation, we were able to discern the head spines in specimens YKLP 17215a (Fig. 2), YKLP 17215b (Fig. 3) and YKLP 17219 (Fig. 4).

The presence of tergopleural spines stands out as one of the most distinctive morphological characteristics distinguishing *T. longicaudata* from other Chengjiang euarthropods (Additional file 1: Fig. S3). In our *μ*CT analyses, these spines are consistently observable in all specimens, appearing at first sight detached from the tergites. While this might initially suggest that they do not represent the lateral boundaries of the tergites but rather isolated structures emerging from a “pleural” and thus not a “dorsal” region, a closer examination reveals a uniform backward folding of these spines across all specimens. Moreover, the fossil impressions clearly indicate that these are not singular structures but rather spines protruding from the lateral boundaries of the tergites, as evidenced in specimens such as those depicted in ref. ([29], fig. 20.18a–c).

We propose that the tergites underwent a folding process akin to an accordion during compression, compaction, and fossilization (Additional file 1: Fig. S3). This implies that the tergopleural spines were relatively more resilient structures compared to the dorsal parts of the tergites. Otherwise, some of these spines would likely have been truncated or folded in alternative directions during the compaction and compression process in at least some specimens. Eventually, the original length of these spines and the angle at which they emerged from the tergites cannot be determined from the fossils.

The complete length of the telson, a distinctive anatomical feature in *T. longicaudata*, appears to be nearly fully preserved in specimens YKLP 17215a (Fig. 2) and YKLP 1721b (Fig. 3) and in the restudied holotype part Hz-f-7–228 and counterpart Hz-f-7–229 in our investigation (Fig. 1A, B; Additional file 1: Fig. S1). Among the specimens depicted in the existing literature, the telson is entirely preserved in all referenced specimens except for ?477 ([16], fig. 477). However, the distal-most portion of the telson is not always distinctly visible in the fossils. When observable, it reveals that the telson does not terminate in a sharp spine, as implied by the term “sword-like” from the original description, but rather concludes with a thickened, blunt ending. This interpretation was initially illustrated by ref. ([26], fig. 475]), contradicting the previously suggested “sword-like” tail. Our three-dimensional model (Fig. 7) also reflects this distal portion of the telson, although it is not apparent in our *μ*CT renderings but is discernible in the fluorescent image of the telson in specimen YKLP 17218 (Fig. 6A, inset).

Moreover, a consideration of the refiguring of the “reversed” specimen Hz-f-11–29 (originally in ref. ([17], pl. 9, Fig. 3]), later in ref. ([25], pl. 8, Fig. 2]) reveals that this fossil specimen also exhibits a rather blunt, rounded, thickened distal part of the telson.

The diverse array of morphological characters observed in *T. longicaudata* may indicate a considerable degree of phenotypic plasticity, suggesting a high intraspecific variability within this Cambrian euarthropod. While the absence of certain structures in specific specimens might be attributable to taphonomic biases, the presence of other structures poses a more nuanced challenge. Notably, features such as the blunt ending of the telson, head spines, and lateral spines were not initially documented in the original description. Throughout the literature, the elongate telson emerges as the sole consistent feature across all specimens.

Several studies have previously suggested intraspecific variability in Cambrian euarthropods, as observed in the Chengjiang artiopod *Sinoburius lunaris* [47] and South Chinese trilobites *Duyunaspis* sp. and *Balangia* sp. [48]. However, a more comprehensive understanding of the morphological range within a species necessitates a larger sample size.

It is crucial to acknowledge that some specimens assigned to *T. longicaudata* might potentially represent distinct species. Furthermore, these specimens could also represent different ontogenetic stages of *T. longicaudata* itself or even stages of closely related taxa such as *H. ercaiensis*, *J. multisegmentalis*, or *Y. tenuis* [15].

Of particular interest is the resemblance of the small terminal setae alongside the margin of the rounded, blunt ending of the telson in *T. longicaudata* to the setaceous structures observed in the flap-like telson of *Y. tenuis* ([15], Fig. 6E, F). This observation raises intriguing possibilities, such as *T. longicaudata* representing early ontogenetic stages of *Y. tenuis*, with the telson potentially being resorbed in later stages.

## Conclusions

This investigation contributes to a more comprehensive understanding of one additional member within the distinguished and remarkably preserved Chengjiang biota of South China—specifically, the megacheiran *Tanglangia longicaudata*. Despite the intricate history of depicted specimens and inaccurately assigned labels in the existing literature, we successfully unraveled the ventral and appendicular morphology of this species. *Tanglangia longicaudata* distinguishes itself among the “great appendage” euarthropods through its distinctive head spines, elongate telson, and tergopleural spines, features not observed in any other megacheiran. Our examination revealed the presence of four biramous post-great appendage head appendages in multiple specimens. Additionally, we demonstrated that each biramous trunk appendage comprises a slender, elongated endopodite with seven to ten elements (likely employed in walking) and a leaf-shaped exopodite adorned with fringed setae (presumably utilized in swimming, osmoregulation, and/or breathing). We also identified a smaller, singular structure emerging from the basipodite, potentially representing an exite—a feature recently observed in two other megacheirans. While the morphology of trunk appendages in the species *Tanglangia longicaudata* (excluding the presence of an exite) was previously hypothesized, our study, employing state-of-the-art virtual techniques such as *μ*CT, provided the first empirical evidence based on fossils, underscoring the advantages of modern technology in Chengjiang biota morphology analyses. *Tanglangia longicaudata* remains a rare species, with, as demonstrated, only fewer than a dozen specimens discovered and illustrated in the past three decades. Having more specimens available in the future holds the potential to augment our understanding of this taxon, potentially revealing intraspecific variation, shedding light on ontogenetic changes, or uncovering sexually related dimorphism—a relatively overlooked aspect in the interpretation of fossilized arthropods.

## Methods

### Material

The following eight fossil specimens of *Tanglangia longicaudata* have been investigated in this study: previously published part Hz-f-7–228 and counterpart Hz-f-7–229 of the holotype (Fig. 1A, B, Additional file 1: Fig. S1; see ref. ([17], pl. 9, Fig. 2a, b), as well as YKLP 13917 (Fig. 1C–E; see also ref. ([29], fig. 20.18a). Additional specimens studied here are YKLP 17215a part (Fig. 2) and YKLP 17215b counterpart (Fig. 3), furthermore YKLP 17219 (Fig. 4, Additional file 1: Fig. S2A), YKLP 17217 (Fig. 5, Additional file 1: Figs. S2B, and S3), and YKLP 17218 (Fig. 6, Additional file 1: Fig. S2C).

### Preparation, imaging, and rendering

The fossils were prepared with steel needles to remove the matrix. Macro-photography was then conducted using a Leica DFC500 digital camera connected to a Leica M205C stereo microscope (Leica Microsystems, Wetzlar, Germany). All specimens were scanned with a Micro-X-ray-CT [49]: Xradia 520 Versa (Carl Zeiss X-ray Microscopy, Inc., Pleasanton, USA). Scanning parameters are as follows: YKLP 17215a (beam strength: 70 kV/6 w, no filter, resolution: 10.657 μm, number of TIFF images: 3260); YKLP 17215b (beam strength: 70 kV/6 w, no filter, resolution: 7.153 μm, number of TIFF images: 3627); close-up of the head region in YKLP 17215b (beam strength: 70 kV/6 w, no filter, resolution: 4.606 μm, number of TIFF images: 1726); YKLP 17217 (beam strength: 60 kV/5 w, no filter, resolution: 6.733 μm, number of TIFF images: 3293); YKLP 17218 (beam strength: 70 kV/6 w, no filter, resolution: 6.310 μm, number of TIFF images: 2199); YKLP 17219 (beam strength: 70 kV/6 w, no filter, resolution: 7.314 μm, number of TIFF images: 2292); Hz-f-7–228 (bam strength: 70 kV/6 w, no filter, resolution: 10.79 μm, number of TIFF images: 3693); Hz-f-7–229 (beam strength: 60 kV/5 w, no filter, resolution: 10.83 μm, number of TIFF images: 3593). Radiographs generated from each scan were saved as TIFF stacks and further processed in Drishti (version 2.4) [50]. Screenshots of the 3D models were saved in Drishti 2.4. Brightness and contrast of the images and screenshots were adjusted in Photoshop CC. All figures were assembled in Inkscape vers. 1.2 and exported as PNGs. The 3D reconstructions shown in Fig. 7 were generated in Blender vers. 4.0. The 2D drawings shown in Fig. 8 were created in Inkscape vers. 1.2.

### Supplementary Information

**Additional file 1: Fig. S1. **Drishti renderings of the two *Tanglangia longicaudata* holotype parts. **A** Hz-f-7-228, part. **B** Hz-f-7-229, counterpart. **Fig. S2.** Drishti renderings of the “outer”, dorsal side of three *Tanglangia longicaudata* specimens. **A** YKLP 17219. **B** YKLP 17217. **C** YKLP 17218. **Fig. S3.** Drishti renderings of *Tanglangia longicaudata* specimen YKLP 17217 in ventro-laterally compressed orientation showing details of the triangular shaped, backward folded tergopleural spines (taphonomically biased). **A**, **C**, **E** Drishti rendering with activated shadow renderer. **B**, **D**, **F** Drishti rendering without activated shadow renderer. **A**, **B** anterior oblique view. **C**, **D** anterior oblique view, specimen inclined. **E**, **F** posterior oblique view. Not to scale.

**Additional file 2.** 3D model of *Tanglangia longicaudata*.

## Data Availability

All fossil specimens were collected from Yu’anshan Member of the Chiungchussu Formation, Dazi section of Haikou County in Kunming, Yunnan, China, and have been imaged, CT-scanned, and analyzed at the Yunnan Key Laboratory for Palaeobiology (YKLP). They are housed either at the Yunnan Key Laboratory for Palaeobiology, Yunnan University (YKLP 13917, YKLP 17215a, YKLP 17215b, YKLP 17217, YKLP 17218, YKLP 17219), or at the Yunnan Institute of Geological Survey (Hz-f-7–228 and Hz-f-7–229). The 3D-model of *Tanglangia longicaudata* is available as Additional File 2.

## Abbreviations

1–4: Claw elements 1–4 of great appendage
1–12: Tergopleural spines 1–12
A1: Great appendage
ahs: Anterior head spine
an: Anterior
asc: Anterior sclerite
A*n*: Biramous head appendage *n*
ba(s): Basipodite
Blue arrows: Exites of biramous trunk appendages
ce*n*: Claw element of the great appendage
ee: Elements of the endopodite
en: Endopodite
es: Eye stalk
et: Exite
ex: Exopodite
ey: Eye
hs: Head shield
hy?: Potential hypostome/labrum complex with marginal setae
pe: Peduncle
pe*n*: Peduncle element of the great appendage
phs: Posterior head spine
pl: Palpebral lobe
pos: Posterior
Red arrows: Tergopleurae of trunk tergites
Red asterisks: Biramous head appendages 1–4
se: Setae
Ta: Biramous trunk appendages
te: Telson
t*n*: Tergite *n*
T*n*: Biramous trunk appendage *n*
tps: Tergopleural spines
White arrowheads: Furrows on the dorso-lateral side of head shield
