# Supplementary figures and images for: Unveiling the ventral morphology of a rare early Cambrian great appendage arthropod from the Chengjiang biota of China
Source: BMC Biol. 2024 Apr 29;22:96. doi: 10.1186/s12915-024-01889-y (PMC11057168; doi:10.1186/s12915-024-01889-y)

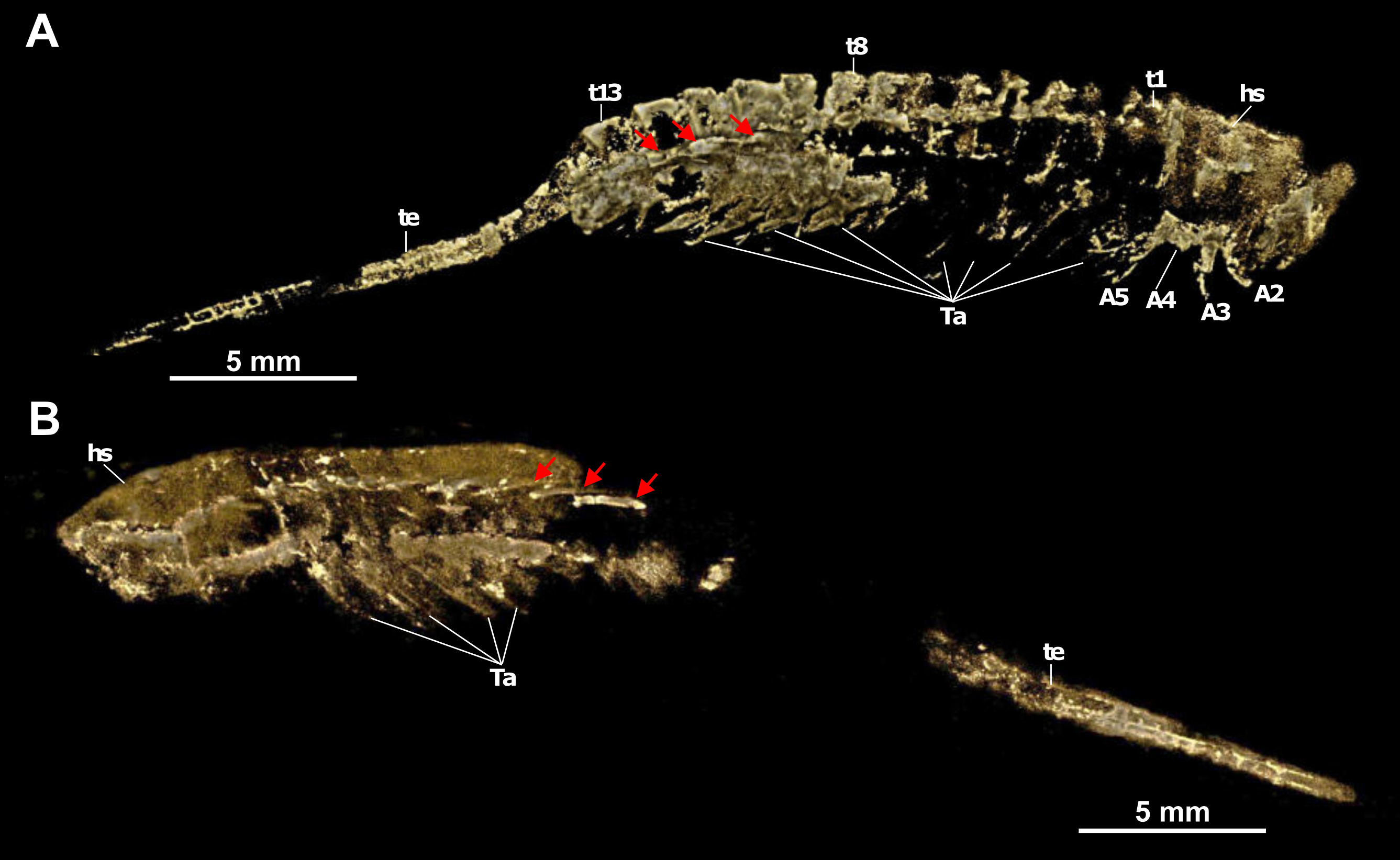

Supplement: Supplementary file 1 — Additional file 1: Fig. S1. Drishti renderings of the two Tanglangia longicaudata holotype parts. A Hz-f-7-228, part. B Hz-f-7-229, counterpart. Fig. S2. Drishti renderings of the “outer”, dorsal side of three Tanglangia longicaudata specimens. A YKLP 17219. B YKLP 17217. C YKLP 17218. Fig. S3. Drishti renderings of Tanglangia longicaudata specimen YKLP 17217 in ventro-laterally compressed orientation showing details of the triangular shaped, backward folded tergopleural spines (taphonomically biased). A, C, E Drishti rendering with activated shadow renderer. B, D, F Drishti rendering without activated shadow renderer. A, B anterior oblique view. C, D anterior oblique view, specimen inclined. E, F posterior oblique view. Not to scale. [file 12915_2024_1889_MOESM1_ESM.zip › ADDITIONAL FILE 1/Fig. S1.png]

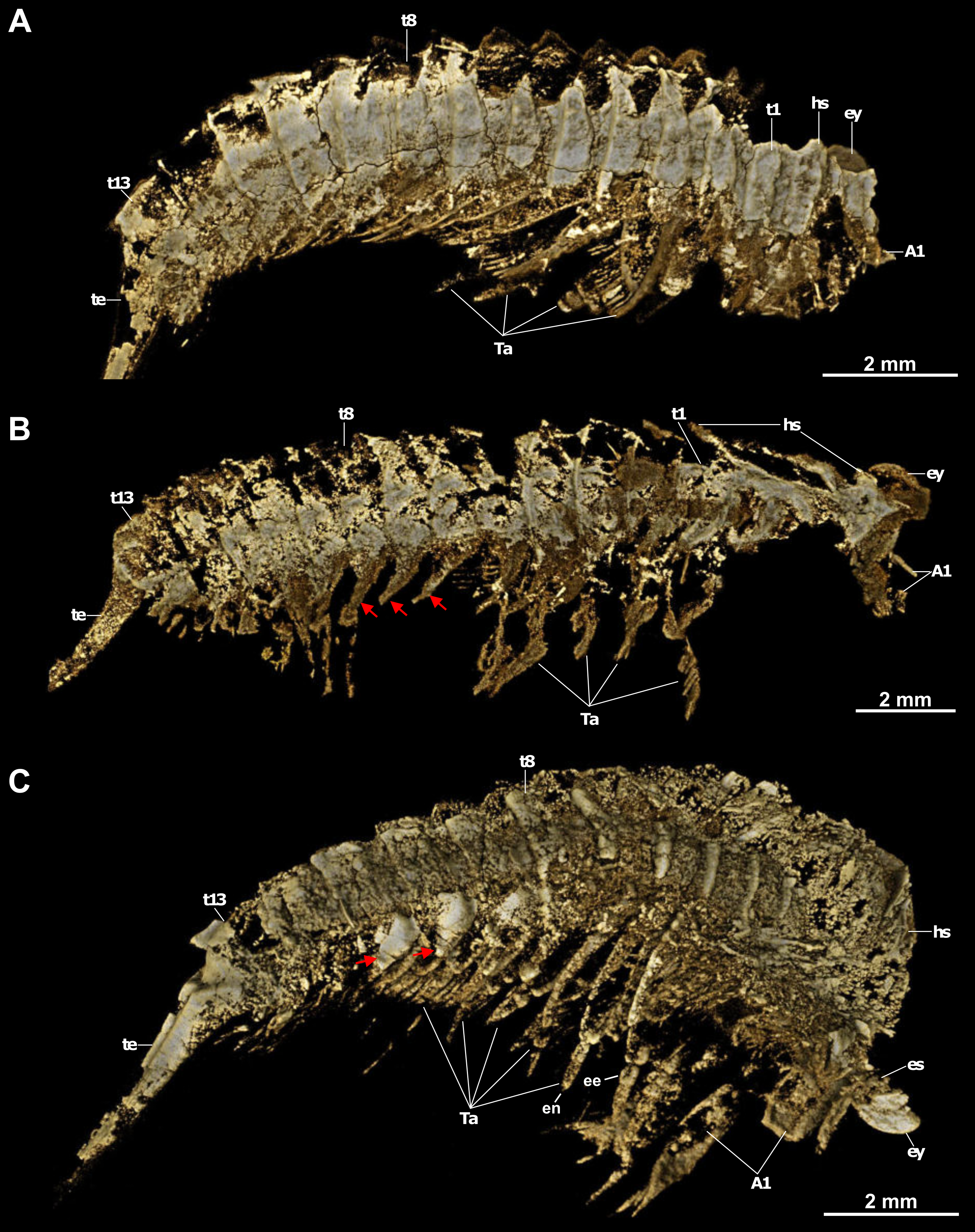

Supplement: Supplementary file 1 — Additional file 1: Fig. S1. Drishti renderings of the two Tanglangia longicaudata holotype parts. A Hz-f-7-228, part. B Hz-f-7-229, counterpart. Fig. S2. Drishti renderings of the “outer”, dorsal side of three Tanglangia longicaudata specimens. A YKLP 17219. B YKLP 17217. C YKLP 17218. Fig. S3. Drishti renderings of Tanglangia longicaudata specimen YKLP 17217 in ventro-laterally compressed orientation showing details of the triangular shaped, backward folded tergopleural spines (taphonomically biased). A, C, E Drishti rendering with activated shadow renderer. B, D, F Drishti rendering without activated shadow renderer. A, B anterior oblique view. C, D anterior oblique view, specimen inclined. E, F posterior oblique view. Not to scale. [file 12915_2024_1889_MOESM1_ESM.zip › ADDITIONAL FILE 1/Fig. S2.png]

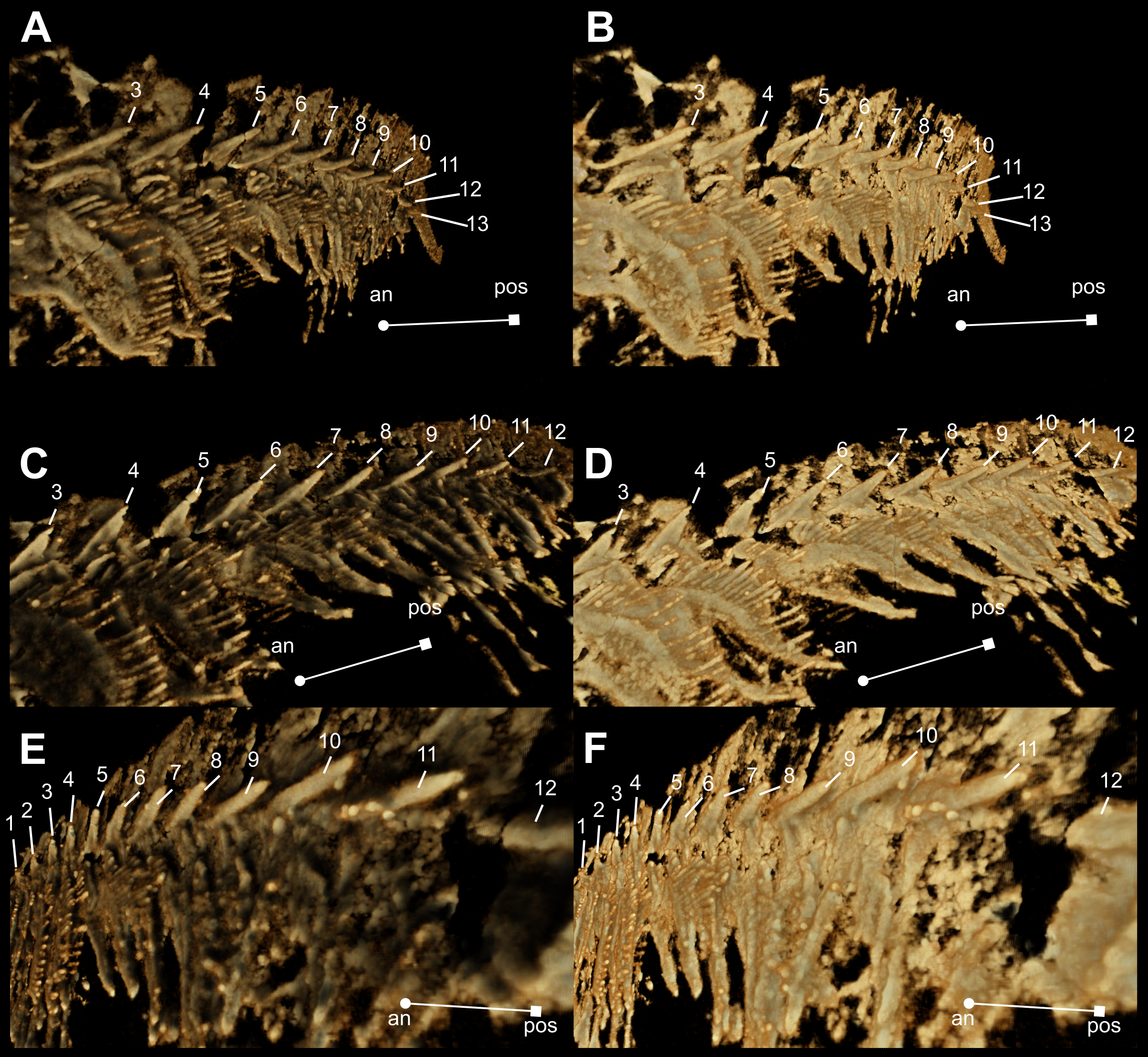

Supplement: Supplementary file 1 — Additional file 1: Fig. S1. Drishti renderings of the two Tanglangia longicaudata holotype parts. A Hz-f-7-228, part. B Hz-f-7-229, counterpart. Fig. S2. Drishti renderings of the “outer”, dorsal side of three Tanglangia longicaudata specimens. A YKLP 17219. B YKLP 17217. C YKLP 17218. Fig. S3. Drishti renderings of Tanglangia longicaudata specimen YKLP 17217 in ventro-laterally compressed orientation showing details of the triangular shaped, backward folded tergopleural spines (taphonomically biased). A, C, E Drishti rendering with activated shadow renderer. B, D, F Drishti rendering without activated shadow renderer. A, B anterior oblique view. C, D anterior oblique view, specimen inclined. E, F posterior oblique view. Not to scale. [file 12915_2024_1889_MOESM1_ESM.zip › ADDITIONAL FILE 1/Fig. S3.png]
